# Outcomes After Proximal Humerus Surgery: Does Regional Anesthesia Usage Matter?

**DOI:** 10.1016/j.jhsg.2025.100920

**Published:** 2026-01-13

**Authors:** Bill Young, Amy L. Ladd

**Affiliations:** Department of Orthopaedic Surgery, Stanford University, Stanford, CA

**Keywords:** Emergency departmentl, Fracture, Opioid, Proximal humerus, Regional anesthesia

## Abstract

**Purpose:**

Regional anesthesia is commonly used for intraoperative pain control during proximal humerus fracture surgery. We hypothesized that patients undergoing proximal humerus fracture surgery who received regional anesthesia would have increased postoperative emergency department (ED) utilization, increased perioperative opioid prescriptions, and greater incidence of persistent postoperative opioid prescriptions compared with those who received general anesthesia.

**Methods:**

We retrospectively identified patients ≥18 years of age with a closed proximal humerus fracture undergoing either open reduction internal fixation (ORIF) or shoulder arthroplasty within 21 days of the fracture. We used International Classification of Diseases (ICD) 9/10 codes to identify patients using an administrative claims database. We categorized and then propensity-score matched patients based on receipt of regional anesthesia during surgery. Our first outcome was any instance of an ED visit within 7 and 30 days after surgery. Secondary outcomes included perioperative opioid prescriptions and the incidence of persistent opioid prescriptions. Multivariable regression models were used to assess the risk of an ED visit or persistent opioid usage based on the receipt of intraoperative regional anesthesia, adjusting for demographics and comorbidities.

**Results:**

In total, 10,580 (33.3%) ORIF patients and 3,299 (33.6%) shoulder arthroplasty patients received regional anesthesia during surgery. Regional anesthesia was associated with an increased 7-day ED visit incidence compared to no regional anesthesia receipt for ORIF patients (3.3% vs 2.3%) and shoulder arthroplasty patients (2.7% vs 1.9%) (*P* < .001 for both). In the 30-day postoperative window, regional anesthesia was associated with an increased incidence of an ED visit for ORIF patients (7.1% vs 5.8%) and shoulder arthroplasty patients (6.6% vs 5.2%) (*P* < .001 for both). Regional anesthesia was also associated with greater perioperative opioid prescriptions across both surgeries (*P* < .05).

**Conclusions:**

Further research should explore interventions for proximal humerus fracture surgery patients who receive regional anesthesia to reduce potentially preventable ED visits and opioid-related adverse events.

**Type of study/level of evidence:**

Therapeutic III.

Proximal humerus fractures are common fractures of the upper extremity, accounting for around 5% to 6% of fractures in adults.^1^ Surgical management of these fractures varies, with open reduction internal fixation (ORIF) and shoulder arthroplasty encompassing the most common approaches. Anesthesia usage for proximal humerus fracture surgery may include regional anesthesia. Regional anesthesia has received increasing interest in orthopedic surgery, with usage increasing in both inpatient and ambulatory surgical settings.^2^^,^^3^ Regional anesthesia has been suggested as a comparatively safer option compared to general anesthesia.^4^^,^^5^ For example, in shoulder arthroplasty patients, general anesthesia was associated with a 40% increase in the odds of prolonged hospitalization and greater odds of systemic medical complications compared with the receipt of regional anesthesia.^6^ Regional anesthesia has also been shown to result in fewer perioperative opioids consumed and shorter postoperative monitoring times compared to patients receiving general anesthesia or both general and regional anesthesia for shoulder arthroscopies.^7^ As a result, there is a proposed benefit of regional anesthesia in improving pain control and reducing postoperative opioid consumption.^8^

However, despite growing interest in regional anesthesia usage, there is still conflicting evidence regarding its benefits on outcomes in orthopedic surgery. One study examining opioid consumption in patients undergoing surgery for wrist fractures and dislocations found an increase in postoperative opioid utilization among patients who received regional anesthesia.^9^ Additionally, patients who received regional anesthesia for wrist fracture surgery showed a greater incidence (12% vs 4%) of unplanned physician visits and severe pain scores in the immediate postoperative period compared to patients who received general anesthesia.^10^ These findings highlight potential challenges related to pain control associated with regional anesthesia receipt. Taken together, there currently exists an opportunity to potentially reduce opioid utilization and prevent unnecessary health care utilization, which we aimed to explore in this study.

In this study, we tested the null hypothesis that patients who received regional anesthesia would not have increased postoperative ED utilization, increased perioperative opioid utilization, and greater incidence of prolonged postoperative opioid usage compared to patients who did not receive regional anesthesia.

## Materials and Methods

In this study, we used the PearlDiver Mariner 170 database (www.pearldiverinc.com; PearlDiver Inc, Colorado Springs, Colorado), an insurance-based claims database with over 170 million patient records. Using the International Classification of Diseases, 9th Revision (ICD-9) and 10th Revision (ICD-10) as well as Current Procedural Terminology (CPT) codes, we retrospectively identified patient records in this database. This database includes longitudinal patient records that are deidentified; thus, this study was exempt from institutional review board approval. This study was conducted in accordance with Strengthening the Reporting of Observational Studies in Epidemiology (STROBE) guidelines.

We retrospectively identified patients ≥18 years of age with a closed proximal humerus fracture from 2011 to 2022 using ICD-9/10 codes (Table S1, available online on the Journal’s website at https://www.jhsgo.org). Patients were required to have active records within the database at least 6 months before and after these index codes. Patients with an open proximal humerus fracture, polytrauma, or a prior opioid use disorder were excluded. These diagnoses were identified using ICD 9/10 codes. We identified patients undergoing either an ORIF or shoulder arthroplasty within 21 days after the fracture using CPT codes (Table S1, available online on the Journal’s website at https://www.jhsgo.org). Patients with incomplete demographic information or with insurance plans other than Medicaid, Medicare, or commercial plans were excluded. Patients undergoing surgical management via an ORIF or shoulder arthroplasty were subsequently categorized into those who received regional anesthesia or those who did not during surgery, which was defined using CPT codes, including single-shot and catheter blocks (Table S1, available online on the Journal’s website at https://www.jhsgo.org). The regional and nonregional anesthesia cohorts were then propensity-score matched to each other based on age, gender, and Charlson Comorbidity Index at a ratio 1:2, respectively. Variables were predefined in the PearlDiver database. This resulted in a final ORIF cohort of 10,580 patients undergoing surgery with regional anesthesia and 21,160 patients without. For the shoulder arthroplasty cohort, this resulted in 3,299 patients undergoing surgery with regional anesthesia and 6,510 patients undergoing surgery without. For both surgery cohorts, we examined demographic characteristics, including age, gender, geographic region in the United States, and insurance plan, as well as rates of comorbidities between those who received and did not receive regional anesthesia (Tables 1 and 2). Comorbidities investigated included asthma, congestive heart failure, coronary artery disease, chronic kidney disease, chronic obstructive pulmonary disease, diabetes mellitus, hypertension, obesity, osteoarthritis, rheumatoid arthritis, tobacco use, alcohol abuse, depression, prior lobectomy, and lung cancer.

**Table 1:** ORIF Matched Cohort Demographics

Variable	Regional Anesthesia (n = 10,580)	No Regional anesthesia (n = 21,160)	*P* Value
Age, n (%)			<.001
50–54	893 (8.4)	1,626 (7.7)	
55–59	1,541 (14.6)	2,761 (13.1)	
60–64	1,943 (18.4)	3,440 (16.3)	
65–69	1,783 (16.8)	3,580 (16.9)	
70–74	1,608 (15.2)	4,022 (19.0)	
75+	2,812 (26.6)	5,731 (27.1)	
Gender, n (%)			.988
Female	8,309 (78.5)	16,615 (78.5)	
Male	2,271 (21.5)	4,545 (21.5)	
Insurance type, n (%)			<.001
Commercial	8,004 (75.6)	13,722 (64.9)	
Medicare advantage	2,039 (19.3)	6,360 (30.0)	
Medicaid managed care	537 (5.1)	1,078 (5.1)	
Region, n (%)			<.001
Midwest	8,004 (75.6)	13,722 (64.9)	
Northeast	2,039 (19.3)	6,360 (30.0)	
South	537 (5.1)	1,078 (5.1)	
West	8,004 (75.6)	13,722 (64.9)	
Comorbidities			
Asthma	2,254 (21.3)	4,116 (19.5)	<.001
Congestive heart failure	1,917 (18.1)	4,569 (21.6)	<.001
Coronary artery disease	3,127 (29.6)	6,685 (31.6)	<.001
Chronic kidney disease	2,057 (19.4)	4,519 (21.4)	<.001
Chronic obstructive pulmonary disease	1,162 (11.0)	2,576 (12.2)	.002
Diabetes mellitus	3,828 (36.2)	8,281 (39.1)	<.001
Hypertension	8,195 (77.5)	16,574 (78.3)	.080
Obesity	4,253 (40.2)	7,370 (34.8)	<.001
Osteoarthritis	5,939 (56.1)	10,780 (50.9)	<.001
Rheumatoid arthritis	813 (7.7)	1,455 (6.9)	.009
Tobacco use	4,865 (46.0)	8,962 (42.4)	<.001
Alcohol abuse	1,640 (15.5)	2,994 (14.1)	.001
Depression	5,197 (49.1)	9,627 (45.5)	<.001
Prior pulmonary resection	15 (0.1)	22 (0.1)	.354
Lung cancer	205 (1.9)	482 (2.3)	.052

**Table 2:** Shoulder Arthroplasty Matched Cohort Demographics

Variable	Regional anesthesia (n=3,299)	No regional anesthesia (n=6,510)	*P* value
Age, n (%)			<.001
50–54	77 (2.3)	110 (1.7)	
55–59	148 (4.5)	264 (4.1)	
60–64	386 (11.7)	510 (7.8)	
65–69	523 (15.9)	1,021 (15.7)	
70–74	827 (25.1)	1,751 (26.9)	
75+	1,338 (40.6)	2,854 (43.8)	
Gender, n (%)			.549
Female	2,748 (83.3)	5,455 (83.8)	
Male	551 (16.7)	1,055 (16.2)	
Insurance type, n (%)			<.001
Commercial	2,158 (65.4)	3,265 (50.2)	
Medicare advantage	1,065 (32.3)	3,107 (47.7)	
Medicaid managed care	76 (2.3)	138 (2.1)	
Region, n (%)			<.001
Midwest	932 (28.3)	1,725 (26.5)	
Northeast	454 (13.8)	1,107 (17.0)	
South	1,478 (44.8)	2,926 (44.9)	
West	435 (13.2)	752 (11.6)	
Comorbidities			
Asthma	658 (19.9)	1,265 (19.4)	.563
Congestive heart failure	871 (26.4)	1,843 (28.3)	.049
Coronary artery disease	1,246 (37.8)	2,492 (38.3)	.638
Chronic kidney disease	915 (27.7)	1,746 (26.8)	.348
Chronic obstructive Pulmonary disease	473 (14.3)	968 (14.9)	.501
Diabetes mellitus	1,457 (44.2)	2,868 (44.1)	.935
Hypertension	2,933 (88.9)	5,784 (88.8)	.959
Obesity	1,524 (46.2)	2,674 (41.1)	<.001
Osteoarthritis	2,386 (72.3)	4,376 (67.2)	<.001
Rheumatoid arthritis	302 (9.2)	542 (8.3)	.179
Tobacco use	1,351 (41)	2,336 (35.9)	<.001
Alcohol abuse	310 (9.4)	504 (7.7)	.006
Depression	1,495 (45.3)	2,705 (41.6)	<.001
Prior pulmonary resection	<11∗	12 (0.2)	N/A
Lung cancer	61 (1.8)	148 (2.3)	.108

∗ Counts less than 11 not reported.

We compared postoperative outcomes for patients who received either regional anesthesia or not, specifically ED visit postoperative utilization and postoperative opioid prescriptions. For ED utilization, we recorded any instance of an ED visit in the 7- and 30-day postoperative windows. We defined perioperative opioid prescription as the mean total amount and mean daily amount of morphine milligram equivalents prescribed within 30 days before and after fracture surgery. We also investigated the incidence of persistent opioid prescription, which was defined as any opioid prescription within 90 to 180 days after surgery among patients prescribed opioids perioperatively, which is consistent with past literature.^9^ For example, if a patient was prescribed opioids immediately following surgery and continued to receive opioid prescriptions 90 days later, this was defined as persistent prescriptions.

### Statistical analysis

We used the Student *t* test to compare between continuous variables and the chi-squared test to compare between categorical variables. Multivariate regression models were used to assess the risk of postoperative ED utilization based on regional anesthesia receipt, adjusting for age, gender, geographical region, insurance plan, service location, and comorbidities. The risk of persistent opioid prescriptions because of regional anesthesia receipt was also assessed using a multivariate regression model, adjusting for the same demographic variables. Comorbidities that differed significantly in proportion between regional and nonregional cohorts based on an alpha of 0.05 were included in regression models. We also performed a subanalysis among only the patients who received regional anesthesia, comparing outcomes between those who received regional anesthesia via single-shot or catheter blocks. We assessed absolute risk using the number needed to harm (NNH) or the number of patients needed to cause one additional adverse event.

## Results

In total, 10,580 (33.3%) ORIF patients and 3,299 (33.6%) shoulder arthroplasty patients received regional anesthesia during surgery (Tables 1 and 2). The mean age for patients who received regional anesthesia in the ORIF cohort was 62.6 ± 11.6 and 63.4 ± 11.7 for those who did not receive regional anesthesia. The mean age for patients who received regional anesthesia in the total shoulder arthroplasty (TSA) cohort was 71.5 ± 7.4 and 72.3 ± 6.9 for those who did not receive regional anesthesia. Of the ORIF patients who received regional anesthesia, 2,977 (28.1%) patients were treated inpatient; however, for those who did not receive regional anesthesia, 8,299 (39.2%) patients were treated inpatient (*P* < .001). Of the TSA patients, inpatient management rates were 2,274 (68.9%) versus 5,101 (78.4%) patients, respectively (*P* < .001).

Seven-day ED visit incidence between those receiving and not receiving regional anesthesia was 349 (3.3%) versus 488 (2.3%) for ORIF patients, representing an absolute increase of 1.0%, and 89 (2.7%) versus 124 (1.9%) for shoulder arthroplasty patients, representing an absolute increase of 0.8% (*P* < .001 for both) (Table 3). The NNH was 101 patients for ORIF surgery and 127 patients for TSA. Of these 7-day visits, visits in the immediate 48-hour period following surgery were common, with 101 (28.9%) versus 123 (25.2%) patients presenting to the ED in this period following ORIF surgery, and 27 (32.6%) versus 27 (21.8%) patients following TSA surgery. Thirty-day ED visit incidence was 754 (7.1%) versus 1,224 (5.8%) for ORIF patients, representing an absolute increase of 1.3%, and 217 (6.6%) versus 338 (5.2%) for shoulder arthroplasty patients, representing an absolute increase of 1.4% (*P* < .001 for both). The NNH was 74 patients for ORIF surgery and 72 patients for TSA. In the multivariable analysis, regional anesthesia was associated with an increased 7-day ED visit risk for ORIF patients (odds ratio (OR) 1.39, 95% confidence interval (CI): 1.25–1.56, *P* < .001, NNH: 115) (Table 4). In the 30-day postoperative window, regional anesthesia was associated with an increased ED visit risk for ORIF patients (OR 1.24, 95% CI: 1.15–1.34, *P* < .001, NNH: 77) and shoulder arthroplasty patients (OR 1.16, 95% CI: 1.00–1.34, *P* = 0.049, NNH: 128).

**Table 3:** Breakdown of ED Visits Within 7 and 30 Days After Surgery

Number of ED Visits	ORIF		TSA	
	Regional Anesthesia	No regional Anesthesia	Regional Anesthesia	No Regional Anesthesia
7-day ED visit, n (%)	349 (3.3)	488 (2.3)	89 (2.7)	124 (1.9)
30-day ED visit, n (%)	754 (7.1)	1224 (5.8)	217 (6.6)	338 (5.2)

**Table 4:** Multivariate Regression for Risk of 7- and 30-day ED Visits Based on Receipt of Regional Anesthesia During Surgery

Outcome	Odds Ratio (95% CI)	*P* Value
7-day ED visit		
ORIF	1.39 (1.2–51.56)	<.001
Shoulder arthroplasty	1.18 (0.93–1.49)	.170
30-day ED visit		
ORIF	1.24 (1.15–1.34)	<.001
Shoulder arthroplasty	1.16 (1.00–1.34)	.049

Patients undergoing regional anesthesia for ORIF procedures received greater mean perioperative opioid prescriptions compared to patients not undergoing regional anesthesia (*P* < .001) (Table 5). The regional anesthesia cohort also had an increase in the absolute incidence of persistent opioid prescription rates compared to patients who did not receive regional anesthesia by 1.4% (13.9% vs 12.5%; *P* < 0.001). Patients undergoing regional anesthesia for TSA procedures received greater perioperative opioid prescriptions compared to those who did undergo regional anesthesia (*P* < .05) (Table 5). In the multivariable analysis, regional anesthesia was associated with a greater risk of persistent opioid prescriptions for ORIF patients (OR 1.10, 95% CI: 1.06–1.15, *P* < .001, NNH: 92) (Table 6).

**Table 5:** Amounts of Opioids Prescribed Between 30 days Before and After Proximal Humerus Surgery, and Persistent Opioid Prescription

Outcome	ORIF		*P* value	TSA		*P* value
	Regional anesthesia (n = 10,580)	No regional anesthesia (n = 21,160)		Regional anesthesia (n = 3,299)	No regional anesthesia (n = 6,510)	
Total MMEs per patient, mean (SEM)	872.1 (10.2)	836.8 (7.4)	.006	723.3 (17.2)	692.9 (9.9)	<.001
Total MMEs per patient per day, mean (SEM)	129.4 (1.1)	120.3 (0.8)	<.001	105.4 (1.7)	99.9 (1.1)	.005
n, % persistent opioid prescription	1,472 (13.9)	2,648 (12.5)	<.001	338 (10.2)	716 (11.0)	.254

MMEs, morphine milligram equivalents; SEM, standard error of the mean.

**Table 6:** Multivariate Regression for Risk of Persistent Opioid Prescription Based on Receipt of Regional Anesthesia During Surgery

Outcome	Odds Ratio (95% CI)	*P* Value
Persistent opioid prescription		
ORIF	1.10 (1.06–1.15)	<.001
Total shoulder arthroplasty	0.93 (0.86–1.01)	.092

In the subanalysis comparing the type of regional anesthesia, single-shot block was associated with a greater risk of persistent opioid prescriptions compared to catheter block in the ORIF cohort (Table S2, available online on the Journal’s website at https://www.jhsgo.org). In contrast, shoulder arthroplasty patients undergoing single-shot block had a lower risk of persistent opioid prescriptions compared with patients undergoing catheter block.

## Discussion

In this study, we found that regional anesthesia is associated with an increased incidence of both 7-day and 30-day postoperative ED utilization in patients undergoing either ORIF or shoulder arthroplasty surgery for proximal humerus fractures. Furthermore, regional anesthesia is a risk factor for greater perioperative opioid prescriptions and prolonged opioid prescriptions in patients undergoing ORIF surgery. Shoulder arthroplasty patients who received regional anesthesia were more likely to receive greater perioperative opioid prescriptions. These findings highlight an opportunity to improve pain following regional anesthesia to reduce health care utilization and improve patient outcomes after proximal humerus surgery.

Currently, there is conflicting evidence on how regional anesthesia influences orthopedic surgery postoperative outcomes. For postoperative health care resource utilization, one study found that regional anesthesia receipt for wrist fracture surgery was associated with greater rates of unplanned health care utilization in the immediate postoperative period.^10^ These findings correlate with the increased rates of all-cause ED visits seen in patients who received regional anesthesia in our study. However, in contrast with our findings, regional anesthesia has been shown to shorten recovery after shoulder surgery and also decrease intraoperative opioids.^7^^,^^11^ Another study found that for patients undergoing surgical intervention for wrist fractures and dislocations, regional anesthesia receipt was associated with greater rates of 30-day ED visits and increased postoperative opioid prescriptions.^9^ Interestingly, lower rates of prolonged opioid prescription were observed among regional anesthesia patients. Our study found increased postoperative prescriptions among patients who received regional anesthesia for both ORIF and TSA surgeries. However, ORIF surgeries with regional anesthesia were associated with increased prolonged opioid prescription, whereas TSA surgeries were not associated with rates of prolonged opioid prescription in our study. Taken together, these findings suggest that the relationship between anesthesia type and opioid prescription may vary with surgery type and postoperative setting. This is supported by the findings that in patients undergoing distal radius fracture surgery, regional anesthesia is associated with lower amounts of inpatient opioid utilization but greater amounts of outpatient opioid utilization, whereas patients undergoing proximal humerus fracture surgery demonstrate both increased inpatient and outpatient opioid consumption.^12^^,^^13^

Regional anesthesia was also demonstrated in our study to increase perioperative opioid prescriptions, which may be related to pain control. Regional anesthesia has been shown to demonstrate greater rates of rebound pain, or acute pain after a nerve block has worn off.^14^ In a study of patients undergoing distal radius fracture surgery, usage of a brachial plexus nerve block was associated with better pain scores 2 hours postoperatively; however, worse pain scores are noted at 12 and 24 hours postoperatively.^15^ This is further supported by a study by Loewenstein et al,^16^ who found that peripheral nerve blocks for upper extremity surgery were associated with increased rates of 1-week postoperative ED visits, and over 50% of block patients reported pain as the principal reason for their visit. Additionally, another study found that pain was the most common avoidable diagnosis for unplanned ED visits after a rotator cuff repair.^17^ Increased pain may be a contributory mechanism driving the increased ED visit incidence seen among patients who received regional anesthesia. Another potential mechanism for our findings is the proportion of patients treated inpatient versus outpatient. Inpatient management, which was more common in those who did not receive regional anesthesia, may have allowed for improved pain control immediately postoperatively compared to those treated outpatient and therefore a reduced need for ED visits. However, increased odds of ED visits and persistent opioid prescriptions were seen with regional anesthesia receipt despite adjusting for service location, suggesting that there may be additional factors driving these trends.

Future studies should investigate the impact of regional anesthesia usage on patient outcomes and specifically whether addressing rebound pain reduces unplanned utilization of emergency departments and persistent opioid prescriptions. Prescribing practices should also be addressed by implementing institutional pain protocols that are based on multimodal pain management, which has been successful in total joint arthroplasty in reducing postoperative opioid prescriptions.^18^ Integrating nonopioid strategies into pain control regimens may reduce persistent opioid prescriptions in patients who receive regional anesthesia.^19^ Other strategies include preoperative counseling or provider education training, which should be tailored toward providers, as providers may have traditional versus modern approaches to pain control.

Our study found that rates of regional anesthesia usage were around 33% in proximal humerus fracture surgeries. One previous study examining 380 patients undergoing proximal humerus fracture surgery found that the rate of regional anesthesia receipt was 73%.^13^ Notably, this study was conducted at a single institution, which our study expands on by examining rates at the national level. Given that there is limited research on rates of regional anesthesia receipt in proximal humerus fracture surgery, further research should be done to validate the rates demonstrated in our study. Furthermore, in our study, single-shot block was associated with increased odds of prolonged opioid prescription compared with continuous catheter in patients undergoing ORIF surgery; however, the inverse was seen in patients undergoing TSA. Regional anesthesia for proximal humerus fracture surgery is predominantly single-shot interscalene blocks.^20^ These blocks have been shown to provide effective pain relief for up to 8 hours after proximal humerus fracture surgery; however, when compared to continuous catheter-based blocks, they provide less extended pain relief beyond 48 hours and have a shorter time to first analgesic request.^21^^,^^22^ Further research is needed to assess the optimal block in patients receiving regional anesthesia and to characterize complication rates.

There are several limitations to our study. First, this study relies on accurate coding with the claims database. Patients may have had miscoded records, leading to inaccurate data analyses. Our study relied on the assumption that patient records were accurately coded in the database. Second, we assessed any instance of an emergency department visit postoperatively, and did not subclassify those directly because of the surgery or reason for ED visit. However, this was done to include any ED visit potentially attributed to a patient’s surgery, both directly or indirectly. We were also unable to assess granular details regarding ED visits, including timing, presenting complaint, and care delivered during the visit. Furthermore, patients may have presented to EDs or urgent cares that were not captured in the database, potentially underestimating ED utilization. Third, we did not measure the dosing or frequency of opioid prescriptions across the postoperative windows. This may have masked trends regarding the timeline of opioid prescribing between cohorts. Additionally, because opioid prescription was defined through prescription records, we were unable to account for other methods of pain control that patients may have used, such as over-the-counter medications, or whether patients actually used these prescribed opioids. Therefore, the findings in our study may reflect opioid prescribing practices of providers greater than true patient opioid consumption. Finally, our study did not include liposomal bupivacaine as a form of regional anesthesia; future studies should broaden our analyses of types of regional anesthesia.

Overall, this study demonstrates that regional anesthesia receipt in patients undergoing surgery for a proximal humerus fracture is associated with greater rates of postoperative ED utilization and prolonged opioid prescription. These findings highlight regional anesthesia as a potential risk factor for increased rebound pain, which may lead to increased ED utilization and opioid prescriptions.

## Conflicts of Interest

The authors have no relevant conflicts to disclose.
