# Supplementary material for: Outcomes After Proximal Humerus Surgery: Does Regional Anesthesia Usage Matter?
Source: J Hand Surg Glob Online. 2026 Jan 13;8(2):100920. doi: 10.1016/j.jhsg.2025.100920 (PMC12828752; doi:10.1016/j.jhsg.2025.100920)
Supplement: Supplementary Table 1 [file mmc1.docx]

Table S1. Code Definitions

| **Definition** | **ICD9/10, CPT Codes** |
| --- | --- |
| Closed proximal humerus fracture | ICD-9-D-81200:ICD-9-D-81203, ICD-9-D-81209,ICD-10-D-S42201A, ICD-10-D-S42202A, ICD-10-D-S42209A, ICD-10-D-S42211A, ICD-10-D-S42212A, ICD-10-D-S42213A, ICD-10-D-S42214A, ICD-10-D-S42215A, ICD-10-D-S42216A, ICD-10-D-S42221A, ICD-10-D-S42222A, ICD-10-D-S42223A, ICD-10-D-S42224A, ICD-10-D-S42225A, ICD-10-D-S42226A, ICD-10-D-S42231A, ICD-10-D-S42232A, ICD-10-D-S42239A, ICD-10-D-S42241A, ICD-10-D-S42242A, ICD-10-D-S42249A, ICD-10-D-S42251A, ICD-10-D-S42252A, ICD-10-D-S42253A, ICD-10-D-S42254A, ICD-10-D-S42255A, ICD-10-D-S42256A, ICD-10-D-S42261A, ICD-10-D-S42262A, ICD-10-D-S42263A, ICD-10-D-S42264A, ICD-10-D-S42265A, ICD-10-D-S42266A, ICD-10-D-S42271A, ICD-10-D-S42272A, ICD-10-D-S42279A, ICD-10-D-S42291A, ICD-10-D-S42292A, ICD-10-D-S42293A, ICD-10-D-S42294A, ICD-10-D-S42295A, ICD-10-D-S42296A |
| Open proximal humerus fracture | ICD-9-D-81210:ICD-9-D-81213, ICD-9-D-81219, ICD-10-D-S42201B, ICD-10-D-S42202B, ICD-10-D-S42209B, ICD-10-D-S42211B, ICD-10-D-S42212B, ICD-10-D-S42213B, ICD-10-D-S42214B, ICD-10-D-S42215B, ICD-10-D-S42216B, ICD-10-D-S42221B, ICD-10-D-S42222B, ICD-10-D-S42223B, ICD-10-D-S42224B, ICD-10-D-S42225B, ICD-10-D-S42226B, ICD-10-D-S42231B, ICD-10-D-S42232B, ICD-10-D-S42239B, ICD-10-D-S42241B, ICD-10-D-S42242B, ICD-10-D-S42249B, ICD-10-D-S42251B, ICD-10-D-S42252B, ICD-10-D-S42253B, ICD-10-D-S42254B, ICD-10-D-S42255B, ICD-10-D-S42256B, ICD-10-D-S42261B, ICD-10-D-S42262B, ICD-10-D-S42263B, ICD-10-D-S42264B, ICD-10-D-S42265B, ICD-10-D-S42291B, ICD-10-D-S42292B, ICD-10-D-S42293B, ICD-10-D-S42294B, ICD-10-D-S42295B, ICD-10-D-S42296B |
| Open reduction internal fixation | CPT-23615 |
| Total Shoulder Arthroplasty | CPT-23472 |
| Regional Anesthesia | CPT-64415, CPT-64416, CPT-64417, CPT-64418 |
| Emergency Department Visit | CPT-99281, CPT-99282, CPT-99283, CPT-99284, CPT-99285 |

ICD-9/10 = International Classification of Diseases, Ninth or Tenth Revision. CPT = Current Procedural Terminology.
