# Supplementary material for: Outcomes After Proximal Humerus Surgery: Does Regional Anesthesia Usage Matter?
Source: J Hand Surg Glob Online. 2026 Jan 13;8(2):100920. doi: 10.1016/j.jhsg.2025.100920 (PMC12828752; doi:10.1016/j.jhsg.2025.100920)
Supplement: Supplementary Table 2 [file mmc2.docx]

Table S2. Multivariate regression for risk of 7- and 30-day ED visits and persistent opioid prescription based on receipt of regional anesthesia type during surgery

| **Outcome** | **Odds Ratio [95% CI}**  Reference: Catheter block | **p-value** |
| --- | --- | --- |
| 7-day ED visit  ORIF  Shoulder arthroplasty | 1.29 [0.94-1.82]  1.11 [0.64-2.09] | 0.132  0.339 |
| 30-day ED visit  ORIF  Shoulder arthroplasty | 1.07 [0.87-1.34]  0.93 [0.67-1.32] | 0.540  0.661 |
| Persistent opioid prescription  ORIF  Shoulder arthroplasty | 1.26 [1.12-1.43]  0.66 [0.55-0.81] | <0.001  <0.001 |
